# Development of a protocol for standardized use of a water-soluble contrast agent with polyethylene glycol in post-mortem CT angiography

**DOI:** 10.1007/s00414-024-03218-y

**Published:** 2024-04-03

**Authors:** G. M. Bruch, S. Grabherr, C. Bruguier, F. T. Fischer, R. Soto, V. Magnin, P. Genet

**Affiliations:** 1 Institut Für Rechtsmedizin, Ludwig-Maximillians-Universität München, Nussbaumstr. 26, D – 80336, Munich, Deutschland; 2 Centre Universitaire Romand de Médecine Légale, Centre Hospitalier Universitaire Vaudois, Chemin de La Vulliette 4, CH - 1000 Lausanne 25, Schweiz; 3 Université de Genève, Rue du Général-Dufour 24, CH - 1211 Geneva 4, Schweiz; 4 Centre Universitaire Romand de Médecine Légale, Hôpitaux Universitaires de Genève, Rue Michel-Servet 1, CH – 1211, Geneva 4, Schweiz

**Keywords:** PEG, Polyethylenglycol, PMCTA, Postmortem CT Angiography, Protocol

## Abstract

Computed tomography angiography (PMCTA) is increasingly used in postmortem cases. Standardized validated protocols permit to compare different PMCTA images and make it more easily to defend a case in court. In addition to the well-known technique by Grabherr et al. (2011) which is using paraffin oil as a carrier substance, water-soluble polyethylene glycol 200 (PEG200) can be used in combination with the contrast agent Accupaque® 300. As to date, there exists no standardized protocol for the use of this contrast agent mixture, the aim of this study was to develop a protocol using it. Between 2012 and 2022, 23 PMCTA with PEG200 and Accupaque®300 were performed at the University Centre of Legal Medicine Lausanne (Switzerland) and the Institute of Forensic Medicine Munich (Germany). The images obtained were evaluated regarding the opacification of the vessels and possible artefacts. The best image quality was obtained with a mixing ratio of 1:15 (Accupaque®300:PEG200) and a perfusion volume of 1000 ml in the arterial, 1400 ml in the venous and 350 ml in the dynamic phase. The infusion rates described by Grabherr et al. were confirmed for the three phases. Overall, the opacification of the vessels was diagnostically sufficient. In 13 cases no opacification of the right coronary artery was observed due to a stratification artefact. By using the PMCTA protocol with PEG200 as a carrier, a good overall image quality can be achieved. This protocol offers the possibility to standardize PMCTA with PEG200.

**Supplementary Information:**

The online version contains supplementary material available at 10.1007/s00414-024-03218-y.

## Introduction

After Roentgens discovery of the X-rays 1895, the first attempt of post-mortem (pm) angiographies were performed in the middle of the last century [1, 2]. The vasculature of cadavers was already examined using for example plastiline, diluted with paraffin oil or muscilage combined with dyes (cobalt blue, ultramarine, chrome yellow) and various other substances, most notable described by Schoenmackers et al. [2]. Since then, the field of forensic pm radiology has developed steadily and rapidly, starting about 15 years ago with the first forensic pm computed tomography angiographies (PMCTA) [3, 4]. Over the last two decades, imaging has become increasingly important in forensic medicine.

Scientists have therefore tried to find specific carrier substances and compatible contrast agents to perform CT angiographies in a post-mortem setting. PMCTAs have been performed using different techniques, different contrast agents and different carrier substances. Mainly oily substances with different viscosities, but also aqueous solutions such as barium sulphate, telebrix and clinical contrast agents have been tested and described [5].

Since the introduction of PMCTA into the routine work of forensic pathology, the most widely used and published protocol for PMCTA is the so-called multiphase postmortem computed tomography angiography (MPMCTA) developed by Grabherr et al. [6–8]. In this protocol, paraffin oil is used as a carrier substance, which is mixed with a specific contrast agent, Angiofil® (Fumedica, Muri bei Bern, Switzerland.). The two substances are mixed and injected into the body in three different phases using a special machine, Virtangio® (Fumedica, Muri, Bern, Switzerland): first the arterial system is filled with the contrast mixture, then the venous system is filled, followed by an image acquisition after each injection. Finally, a so-called dynamic phase is performed in which an additional amount of contrast is injected simultaneously with the CT acquisition [9, 10].

In contrast to this PMCTA technique, not all PMCTA techniques are published with a recommended standardized protocol, which makes it difficult to compare different angiographic examinations and does not guarantee consistent high quality and quality control. It also limits the reproducibility and therefore the possibility for different forensic institutes to adopt a specific PMCTA technique [3, 11, 12].

One of the carriers commonly used in forensics is the hygroscopic, water-soluble polyethylene glycol (PEG). This carrier substance is usually used in a mixture with a well-known and clinically widely used water-based contrast agent called Accupaque® (GE Healthcare, USA). PEG has been used for many years in medicine (e.g. as Macrogol®, a laxative) and in cosmetics (especially in creams) [7, 8, 13]. Due to its hygroscopic, i.e. water-absorbing properties, PEG is also used in underwater archaeology for conservation purposes [14, 15]. However, PMCTA with PEG and Accupaque® 300 has only been described in the forensic literature as case reports or in combination with other water-soluble substances [16–20]. To our knowledge, no standardized protocol has been published to date.

In order to fill this gap in the standardization and thus comparable use of angiographic fluids, i.e. the combination of carrier substances and contrast agent, we aimed to define a protocol for MPMCTA using PEG200 as the carrier substance.

## Material and Methods

The study was approved by the Ethics Committee of the Canton of Vaud, Switzerland (No. 2022–01989) and by the Ethics Committee of the Ludwig-Maximilians-Universität Munich, Germany (No. 19–438).

A total of 23 angiographies with polyethylene glycol 200 (PEG 200) and Accupaque®300 were performed during the periods of 2012–2013 and 2020–2022. The PM angiographies on the cadavers (♂17, ♀6; mean age 56 years (16–96 years)) were performed prior to medico-legal autopsies, which were performed on behalf of the public prosecutor. The corpses had a mean height of 170 cm (144–192 cm) and a mean weight of 73 kg (48–121 kg), with a body mass index (BMI) of 19.7—39.1 kg/m^2^ (median 24.4 kg/m^2^).

The MPMCTAs were performed at the University Centre of Legal Medicine (CURML), Lausanne, Switzerland, and at the Institute of Legal Medicine, Munich, Germany. Before each angiography, a native CT (Lausanne: until 2014: CT 8 LightSpeed, from 2015 – 30/09/2020: CT 64 LightSpeed, from 01/10/2020: CT HD750 GE Healthcare, Milwaukee, WI, USA; Munich: CT 64 LightSpeed, all GE Healthcare, Milwaukee, WI, USA). Blood samples were taken from the femoral vein for toxicological analysis. Several perfusion parameters were then tested based on the existing protocol by Grabherr et al. The corresponding CT angiography images were evaluated at the different phases in order to assess image quality, vessel opacification and organ enhancement. As suggested by Grabherr et al., the Virtangio® device was used as the injection device. The volume for the different body sizes was not adapted, as in the protocol of Grabherr et al. [6].

After MPMCTA, a complete medico-legal autopsy was performed in each case according to local and international standards.

The selection of the cases took place based on the preautopsy known information. We included cases, were we assumed the causes of death would be natural and cases without obvious signs of putrefaction. Causes of death were natural in 11 cases, mainly of cardiac origin (n = 10; one ruptured splenic artery aneurysm). Non-natural causes of death included two cases of drowning, one case of asphyxia and five cases of polytrauma. In four cases, the cause of death could not be determined after autopsy.

The condition of the corpses with respect to possible decomposition was assessed radiologically using the radio alteration index (RAI) [21]. We found a median RAI of five (range 0–44). Thus, most of the cadavers showed little or no evidence of decomposition.

Computed tomography was performed with the following parameters:

- Field of view 500 mm, voltage 120 kV, modulated current from 200–400 mAs, tube rotation 0.8 s; pitch 0.998; slice thickness 1.25 mm for the arterial and venous phase and, until 30/04/2021, 2,5 mm for the dynamic phase. After that date, also the dynamic phase was performed with a slice thickness of 1.25 mm.

Liquids used:

- Polyethylene glycol (PEG) 200 g/mol (Sigma-Aldrich®, Merck KGaA, Darmstadt, Germany) is a liquid, water-soluble polymer. It is hygroscopic, non-toxic and biodegradable.
- Accupaque® 300 is a clinical contrast agent manufactured by GE Healthcare (GE Healthcare Buchler GmbH & Co.KG, Braunschweig, Germany). It is water soluble and its radiopaque ability is due to its high iohexol (iodine) content.

Before performing the first MPMCTA with a PEG-Accupaque mixture, angiographies were performed on three forensic cadavers (groups 1 and 2) to confirm the suitability of this mixture for forensic postmortem angiography. Following these successful tests, a thorough verification of the mixing ratio for Polyethylene Glycol 200 and Accupaque® 300 was carried out.

We first measured the Hounsfield units (HU) of PEG alone, which were approximately 60. The two substances were then mixed in tubes, in an extracorporeal setting, with different mixing ratios, and the HU of the different mixtures were then measured on the CT images. Accupaque® 300: PEG ratios from 1:8 to 1:24 in eight steps (1:8, 1:10, 1:12, 1:15, 1:18, 1:20, 1:22, 1:24) were tested in tubes in an extracorporeal setting.

**Fig. 1:**
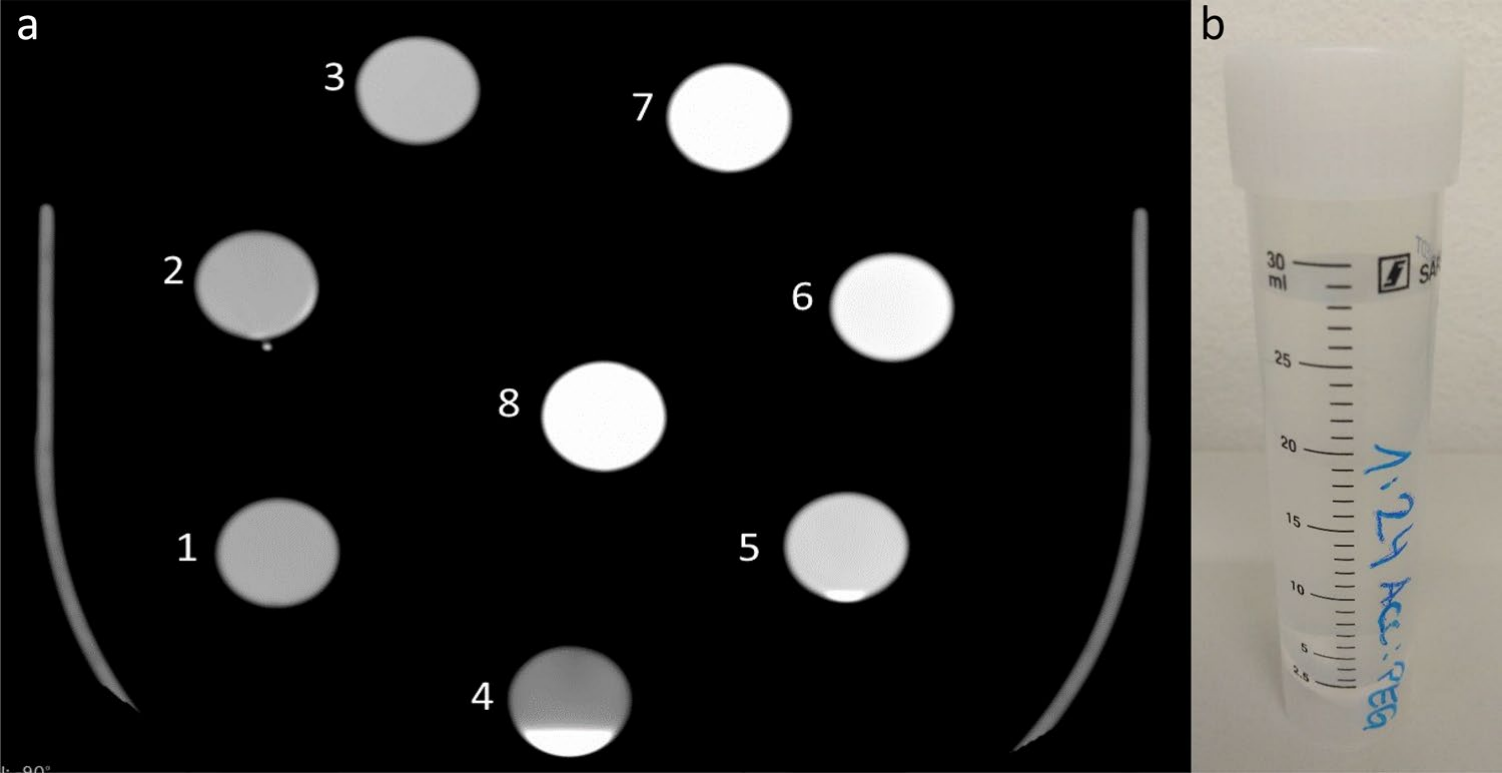
**a**) Tested mixing ratios in the tubes with 120 KVp, WL: 315, WW1229 (1: 1:24 & 520HU; 2: 1:22 & 540HU; 3: 1:20 & 600HU; 4: 1:18 & 330HU above, 4800HU below; 5: 1:15 & 720HU; 6: 1:12 & 880 HU; 7: 1:10 & 1070HU; 8: 1:8 & 1300HU). **b**) An example of the test tube for the 1:24 mixing ratio(No.1)

As a preliminary result, Hounsfield units between 520 (ratio 1:24) and 1300 HU (ratio 1:8) were measured for the specified mixing ratios (Fig. 1a & b). A value of approximately 700 HU was previously determined to represent the best image quality for a voltage of 120 KVp. This value was obtained with a mixing ratio of Accupaque® 300: PEG 200 of 1:15.

Polyethylene glycol itself is non-toxic to humans and nature. Therefore, residues of PEG 200 and also of the clinically used contrast agent Accupaque 300 could be disposed via the respective Institute of Forensic Medicine. There are also no concerns when burying a corpse after PMCTA with PEG-Accupaque, especially as a large part of the contrast agent mixture is already removed from the corpse during the autopsy.

### PM angiography

After a native (no contrast injection) whole-body CT scan a complete external examination of the body was performed. The femoral vessels (artery and vein) were then prepared for cannulation, as described by Grabherr et al. 2008 and 2011 [6, 22].

PEG 200 and Accupaque® 300 were then mixed in the required quantities. In addition, 200 ml of the mixture was always added to fill the pump system. In order to obtain an adequate protocol, we adjusted the injection volumes in the different phases.

The arterial, venous and dynamic phases, together with the CT scans were performed according to the protocol of Grabherr et al. [6]. Finally, we divided our 23 cases into four groups (Table 1).

**Table 1:** Tested angiography parameters

Group	n	Volume (ml) arterial	Volume (ml) venous	Volume (ml) dynamic	Mixing Ratio Accupaque:PEG
1	2	985	985	-	1:9
2	1	1970	1970	-	1:9
3	7	1200	1800	500 (n = 6)*	1:15
4	13	1000	1400	350	1:15

Table 1 Number of angiographies divided into four different groups, according to the injection volumes and the mixtingratio used

*The dynamic phase was omitted in one case due to the rupture of the abdominal aorta seen in the arterial and venous phase

All MPMCTAs were performed with the flow rates established by Grabherr et al. [6]. It was shown that with a flow rate of 800 ml/min for the arterial and venous phase and of 200 ml/min for the dynamic phase a very good image quality could be achieved, and no adaptation seemed to be necessary.

In four cases no dynamic phase was performed. Three of these cases were the first cases in group 1 and 2, one case was a rupture of the abdominal aorta and therefore no dynamic phase was performed.

### Image quality

The images of the different phases were evaluated in terms of image quality. The image quality contains the opacity of the different structures, visualization of previously defined vessels and the possibility of a diagnostic assessability. The images available in DICOM format were analyzed at the CURML, site of Lausanne using the program "AW Server 3.2 Ext. 4.0" and at the Forensic Institute in Munich using the program "Osirix Version 11.0".

To assess the image quality, the filling status of the vessels during the different phases was evaluated separately for the following vessels and organs: superior sagittal venous cerebral sinus (Sinus sag.), transverse venous cerebral sinus (Sinus trans.), bilateral jugular vein (Vv. Jug.), bilateral common carotid artery (Aa. Caro.), right ventricle of the heart (R ventricle), left ventricle of the heart (L ventricle), right coronary artery (RCA), left circumflex coronary artery (LCX), left anterior descending coronary artery (LAD), left pulmonary artery (LPA), right pulmonary artery (RPA), right femoral vein (V. fem. R), right femoral artery (A. fem. R), left femoral vein (V. fem. R), left femoral artery (A. fem. L) and the brachial arteries on both sides (Aa. brach.). The aorta and the vena cava were not evaluated separately because we assumed that these vessels would be filled when the downstream vessels were filled with contrast medium.

Femoral vessels were graded according to the side of cannulation (grading of vessels contralateral to the injection side). All other vessels were graded according to filling status. Some of the vessels were very small in diameter, such as the coronary arteries. Therefore, it was not possible to develop a suitable ROI (region of interest) for all vessels with the same diameter. Instead, the vessels were tracked with the cursor and the Hounsfield units were measured.

Whether the vessel was completely (1), partially (2) or not filled (3) was assessed in each case and for each phase. A vessel was considered 'partially filled' if approximately 50–80% of the vessel was opacified. If the vessel was less than 50% filled, it was recorded as not filled.

**Fig. 2:**
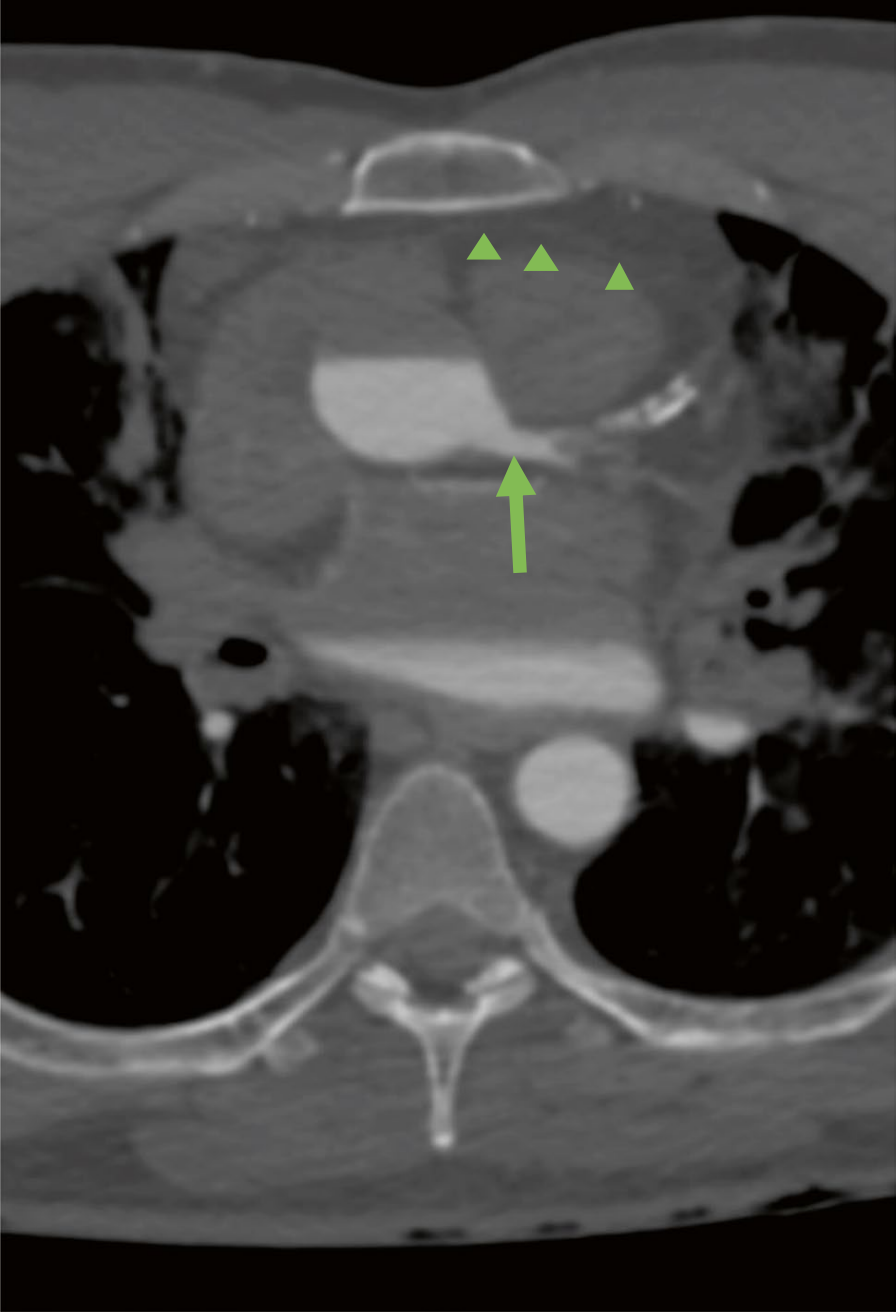
So-called layering in ascending aorta, provokating a non-opacification of the right coronary artery (green arrowheads). The left coronary artery was opacified (green arrow). (axial view; WL: 300, WW: 1500)

Opacification of organs (brain, liver, stomach, spleen, kidneys) was defined as complete (1), partial (2) or not opacified (3). It was documented whether the organ was overfilled [1 +], defined as an overload of contrast agent and leakage from the vessels into the surrounding organ tissue. The presence of a layering artefact was also an aspect of the assessment (Fig. 2). Layering is defined by the presence of two different fluids visible in one vessel. This artefact is an image produced by the fact that the contrast agent mixture used does not mix with the resting blood. In angiographies using PEG200 as a carrier substance the blood is found on top of the contrast agent mixture in the vessels, especially in the ascending aorta (described in the supine position of the body), preventing the filling of the right coronary artery.

### Image quality assessment

Radiological assessment of image quality, filling of the various vessels and enhancement of organ structures was performed by two forensic pathologists with six to 18 years of experience in forensic radiology. For the possible over-enhancement of some organ structures, a ROI of approximately 1 cm^2^ with Hounsfield units of 200 or more was considered significantly overfilled.

## Results

### Accupaque® 300 and Polyethylenglycol 200

Firstly, in an intracorporeal setting, we tested a mixing ratio of Accupaque® 300: PEG 200 of 1:9 in three cases (groups 1 and 2). In these cases, we performed only one arterial and one venous phase, with volumes of 985 ml and 1970 ml, respectively (Table 1). The mixing ratio in this first setting was not optimal, so we decided to test different mixing ratios in an extracorporeal setting (tubes). At a mixing ratio of 1:15, measured values of around 720 HU were obtained in the tubes at a voltage of 120 kV. This seemed ideal.

**Fig. 3:**
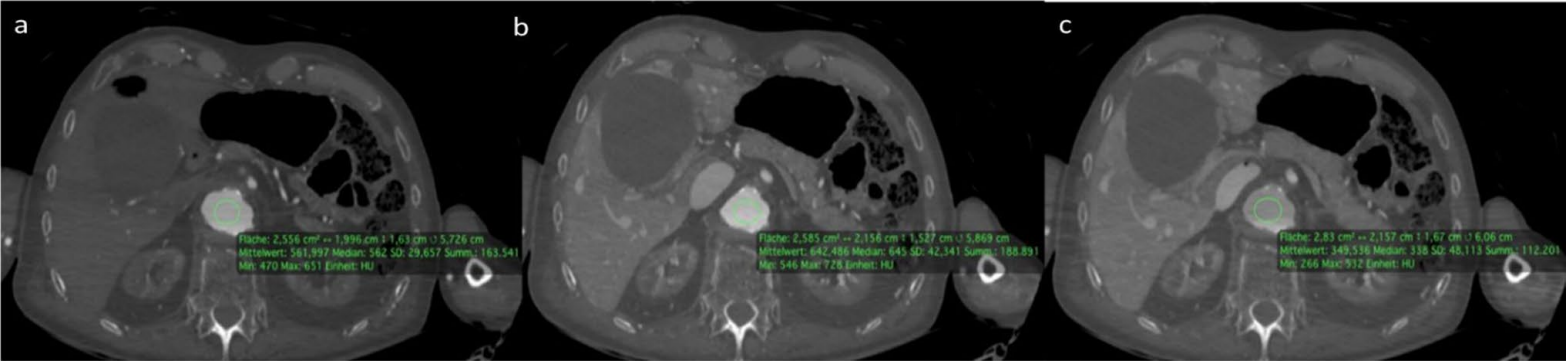
Axial view of the abdomen, each WL: 379, WW: 1536 **a**) After injection of 1200 ml of contrast medium mixture in the arterial phase, the contrast in the abdominal aorta is approximately 560 HU on average. **b**) After injection of another 1800 ml of contrast medium mixture in the venous phase, the contrast in the abdominal aorta is 640 HU on average. **c**) After injection of 500 ml contrast medium mixture in the dynamic phase, the contrast in the abdominal aorta averages only 350 HU

Consequently, the following MPMCTAs were performed with a mixing ratio of Accupaque® 300: PEG 200 of 1:15. However, although this seemed to be optimal in an extracorporeal setting, it turned out not to be the same in an intracorporeal setting (due to the influence of blood in the vessels, which automatically mixes with the contrast agent mixture). After performing MPMCTAs (group 3 in Table 1) with the adapted mixing ratio of 1:15, it was found that this ratio often resulted in about 400–500 HU in the cadavers and not about 720 HU as measured in the tubes. However, we considered the HU measured in the cadavers to be sufficient for a correct assessment of the vascular system and organs (Fig. 3).

After the preliminary tests (group 1 and 2), problems with image quality, mainly due to overfilling artefacts, were assessed. For this reason, we adjusted the injected volumes (group 3 and 4, Table 1).

#### Group 1

In this pre-test group, the arterial vessels were adequately filled. However, the venous system showed significant filling defects. In particular, the venous vessels of the brain (superior sagittal cerebral venous sinus (n = 1) and transverse cerebral venous sinus (n = 2)) and the left ventricle (n = 2) were not completely visualized. The spleen also showed partial opacification in the venous phase (n = 1). The other organs studied (brain, lungs, liver, kidneys) showed good visualization, especially of the arterial organ structures.

The dynamic phase was not performed in these cases because of its preliminary nature.

#### Group 2 and 3

**Fig. 4:**
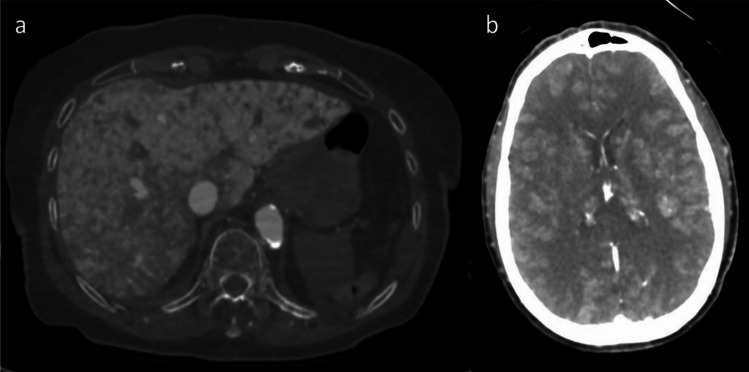
Venous phase after injection of 1200 ml arterial and 1800 ml venous PEG-Accupaque® mixture **a**) Contrast medium leakage into the liver tissue with overfilling (axial view of the abdomen; WL: 249, WW: 1455). **b**) Contrast medium leakage into the white matter of the brain (axial view of the brain; WL: 40, WW: 350)

In group 2 and 3, the arterial and venous vasculature was clearly filled in all anatomical regions examined. Unfortunately, fluid leakage from the vessels into the surrounding tissues was regularly observed. This phenomenon was particularly observed in the venous and dynamic phases. In some cases, the venous phase was overfilled in the liver (n = 5; Fig. 4a), spleen (n = 4) and kidney (n = 3). In three cases, the venous phase also showed contrast leakage into the gastrointestinal tract (Fig. 4a). In two cases, there was also a leakage of the contrast agent mixture into the brain tissue, particularly into the white matter, which appeared to be enhanced (Fig. 4b). In two cases, the contrast agent mixture also (partially) enhanced the wall of the left ventricle of the heart (Fig. 2). One case showed leakage into the subcutaneous fat in the shoulder–neck region.

No dynamic phase was performed in group 2, as this case was part of the pre-test phase. In group 3, overfilling increased during the dynamic phase.

In 6 out of 8 cases in this group, a lack of opacification of the right coronary artery could be seen in the dynamic phase due to the so-called layer artefact in the ascending thoracic aorta (Fig. 2). The overfilling and contrast leakage in the different organ tissues hindered the correct analysis of the vessels and organs.

In summary, overfilling was equally pronounced in both groups.

#### Group 4

**Fig. 5:**
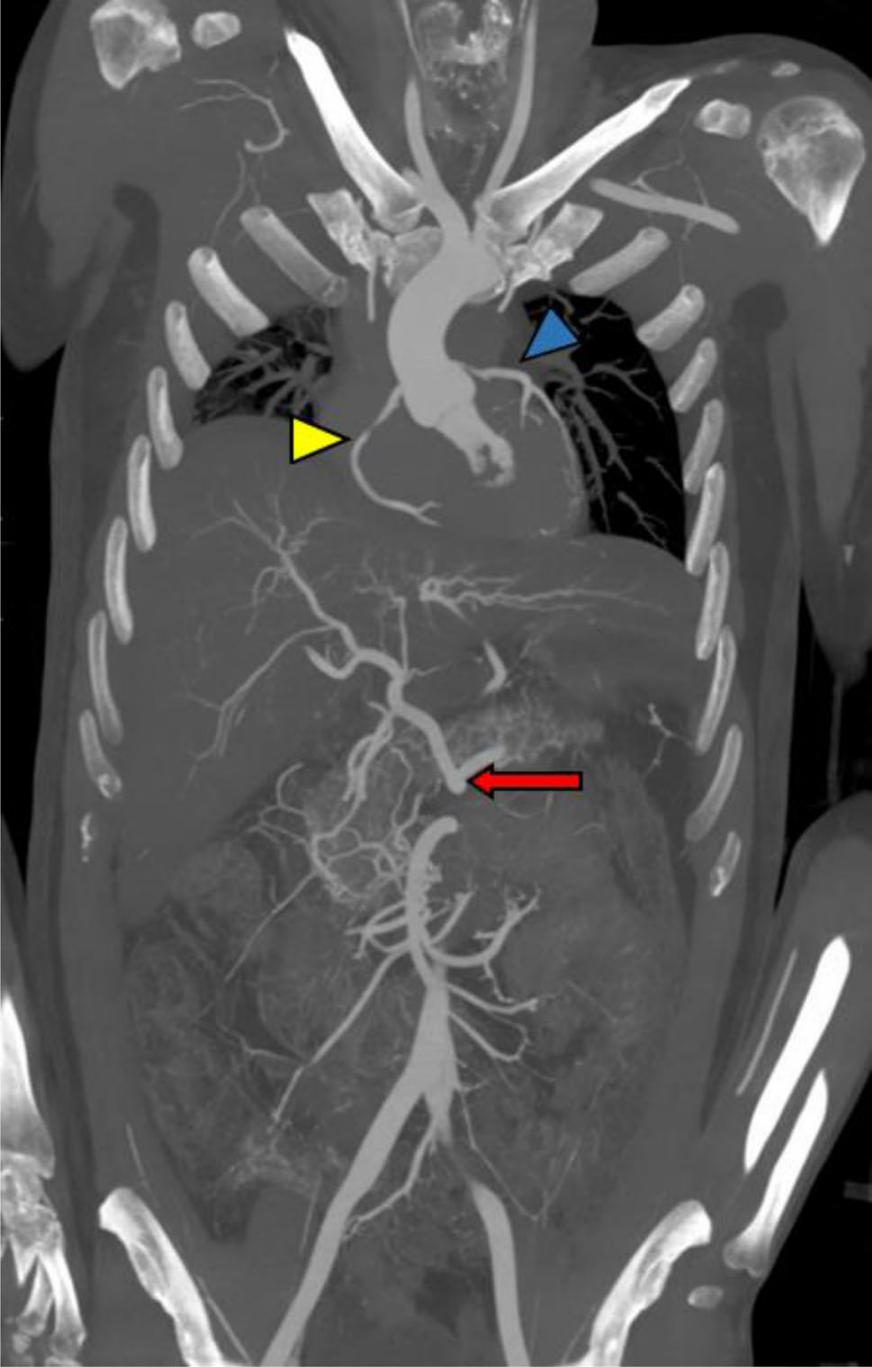
Arterial phase with 1000 ml of contrast agent mixture in coronal view of the thorax and abdomen (MIP reconstruction; WL: 300, WW: 1500): good visualization of the right (yellow arrowhead) and left (blue arrowhead) coronary artery; great visualization of the abdominal arteries and the truncus coeliacus (red arrow)

**Fig. 6:**
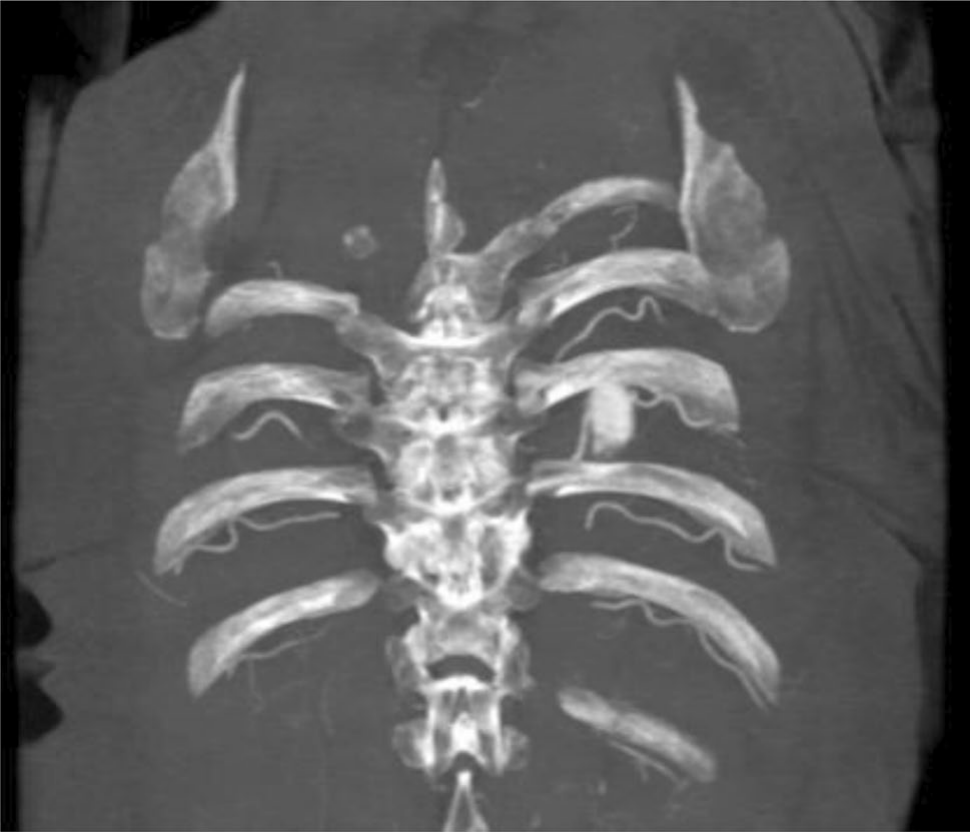
Intercostal arteries after injection of 1000 ml for the arterial phase in Group No. 4 (MIP in coronal posterior view; WL: 300, WW: 1500)

In this group, including 13 PEG based PMCTAs, the arterial and venous system were completely filled and showed good visualization of the vessels and organs examined (Fig. 5). Even vessels such as the intercostal arteries appeared after injection of the arterial phase (Fig. 6). In 8 out of 13 cases, the so-called layer artefact occurred in the ascending thoracic aorta in the arterial phase; in these cases, the right coronary artery was not filled with the contrast mixture. In one case of aortic rupture, the dynamic phase was not performed because contrast leakage had already been detected at the site of rupture.

In the venous phase, there were only three cases of overfilling of the liver and no contrast leakage in the stomach. There was only one case of overfilling of the spleen. The kidney was overfilled in only a few cases (n = 3).

The dynamic phase, with less volume injection than in group 3, also showed better image quality with less overfilling of organs.

**Fig. 7:**
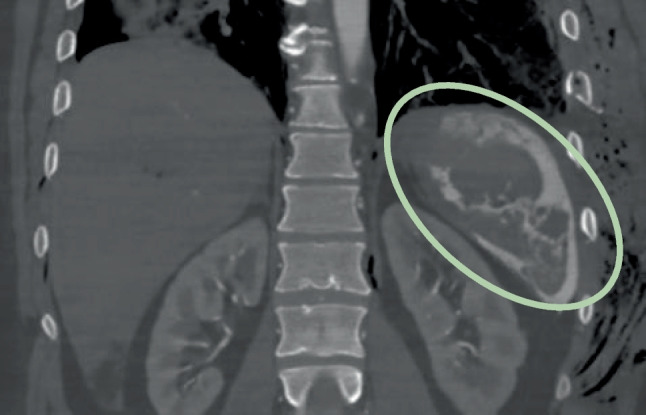
Extravasation of contrast agent mix after injection of 1000 ml during the arterial phase (Group No. 4) due to splenic rupture (coronal view; WL: 300, WW: 1500)

Overall, the image quality was very good and possible pathologies, such as splenic artery rupture (Fig. 7) in the arterial phase, were easily detected.

A detailed evaluation of the vessels and organs in the different angiographic phases at different volumes can be found in Appendix 1.

### Resulting protocol

The final protocol we propose for performing MPMCTA with polyethylene glycol 200 and the water-soluble contrast agent Accupaque® 300 is shown in Table 1. It is based on the results of group 4, as the overall image quality was defined as the best in this group Table 2.

**Table 2:** Resultingprotocol

Phase	Volume	Flow rate*	Mixing Ratio Accupaque:PEG
arterial	1000 ml	800 ml/min	1:15
venouse	1400 ml	800 ml/min	1:15
dynamic	350 ml	200 ml/min	1:15
Pipe filling**	~ 200 ml		
Polyethylenglycol 200			3000 ml
Accupaque®300			200 ml
Total mixture			3200 ml***

The PMCTAs performed with the parameters shown in the resulting protocol had overall the best overall image quality in the evaluation

^*^ According to the protocol of Grabherr et al. 2011

^** ^Note: Pipe filling is an example. The pipe filling depends on the length and width of the pipe system used

^***^ There should be always be mixed more fluid than necessary to avoid air bubbles in the pipe system and later in the vascular system

The 1:15 mixing ratio provides good visualization of vessels and organs throughout the body, while limiting artefacts such as leakage and overfilling of the contrast agent mixture. Vascular occlusions and lesions, haemorrhages and vascular malformations are easily detected. Forensic questions can also be answered easily. The flow rate of 800 ml/min for the arterial and venous phase and 200 ml/min for the dynamic phase can be taken from the Grabherr et al. [6] protocol.

## Discussion

Post-mortem CT angiography (PMCTA) has been a routine procedure in some forensic institutes for many years, but its use is not always standardized. Different procedures and techniques are used with different injection machines and contrast media with different carrier substances. There are few published protocols on the detailed performance of PMCTA, the most widely used being the protocol developed by Grabherr et al. [6–8] called MPMCTA. This protocol uses paraffin oil as a carrier substance and a specific lipophilic contrast agent, Angiofil® (Fumedica, Muri, Bern, Switzerland). An alternative to paraffin oil as a carrier substance is polyethylene glycol 200 (PEG 200), and an alternative to lipophilic contrast agents are water-soluble contrast agents. Each contrast agent and carrier substance has its advantages and disadvantages. It is important to be aware of these as they allow the most appropriate technique to be chosen for each forensic case to be solved. However, the correct use of the different techniques in forensic cases requires standardized protocols for reproducible, reliable and presentable forensic results. To date, only a few case reports and small study groups have been published using PMCTA with PEG 200 and a water-soluble contrast agent. No formal protocol has been published. One of the first to perform and publish PMCTA with PEG 200 was Ross et al. 2008 [19]. This study group performed five postmortem angiographies with 2000 ml of a 1:10 ratio hydrophilic contrast medium-PEG mixture for head and trunk. The problem of the non-contrasted or poorly contrasted right coronary artery has already been described in this publication. The solution was to rotate the cadaver on the CT table. Although this seems to be a practical solution, it was not adequately applicable in our study material, as the cadaver is no longer in the same position as before and the different phases of the PMCTA are no longer optimally comparable.

In 2016, two more papers and case reports [17, 18] were published with angiographies using PEG as a carrier substance. In total, 2500 ml and 1850 ml of fluid were injected to opacify the vessels. These total injection volumes proved to be too low in comparison with our study material. The published case report by Thompsen et al. [17] showed an aortic dissection, so the small vessels e.g. in the brain were not evaluated. Schweitzer et al. [18] showed an opacification of small vessels with a lower contrast mix, but the focus of this evaluation was to compare different pump systems and not to find the optimal volume for PMCTA. Therefore, our aim was to develop a well-functioning protocol for PMCTAs performed with PEG as a carrier substance in combination with a water-soluble and commonly used clinical contrast agent, in our case Accupaque® 300.

### Advantages of a PEG protocol

The recommended protocol with the above parameters showed overall very good image quality. Even small vessels in the brain or abdomen were filled with contrast medium and could be assessed very well. With these settings, good to very good image quality was achieved in most cases for PMCTAs performed with the PEG carrier. It was not necessary to adapt the setting to the body size of most adult corpses or to the state of the corpse (degree of decomposition).

The PEG-PMCTAs are used by forensic pathologists for routine cases that are often presented in court. In court, it is necessary to be able to rely on validated and published protocols to defend the work performed. Our protocol provides such a validation. To our knowledge, no study has been published with such a large number of standardised PEG-PMCTAs. In order to compare different techniques and modalities of PMCTA, it is necessary to have well performed and validated protocols. The existing protocol by Grabherr et al. for angiographies performed with paraffin oil as a carrier substance and the lipophilic contrast agent Angiofil® facilitated the introduction of PMCTA in forensic institutes. The protocol also allowed a large multi-centre study of MPMCTA to be performed using the protocol of Grabherr et al. Further research approaches for post-mortem imaging were developed from this. The development of our protocol for PEG-supported PMCTA was based on this existing protocol, and some of the parameters could be used in the same way. We were even able to find some observations that were already described by Grabherr et al., such as the enhancement of the gastric wall due to accelerated autolysis by gastric juice [10].

It is important to know the advantages and disadvantages of each carrier substance and contrast agent in order to choose the most appropriate type of PMCTA technique for each case. For example, it is essential to have an alternative to the protocol of Grabherr et al., as the oily carrier substance of this protocol is not applicable for certain medico-legal issues [23]. In the case of fat embolism in trauma or medical malpractice, e.g. after hip surgery, a PMCTA with an oily contrast agent mixture would obscure the vital fat embolism on histological examination due to its lipophilic nature [24–26]. The diagnosis of a (fatal) fat embolism is not possible with this type of angiography, but may be possible with a hydrophilic contrast agent mixture. As the lipophilic contrast agent and the fat embolism are indistinguishable in the histological staining required to detect both, fat embolisms in the pulmonary vessels cannot be detected histologically after oil-based angiography.

### Limitations and further research

**Fig. 8:**
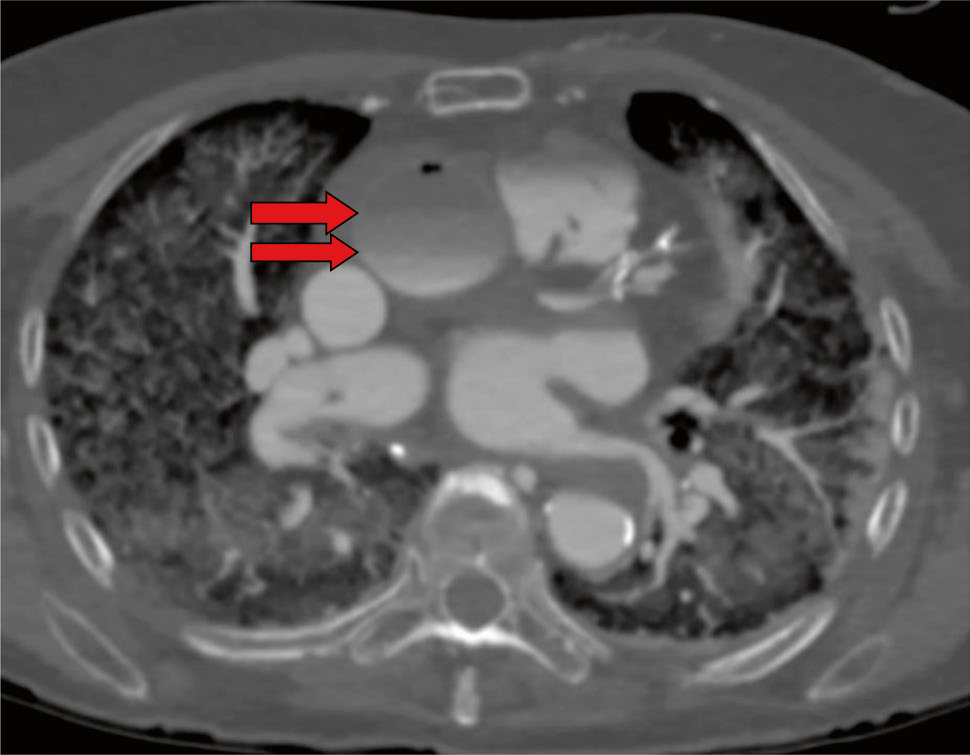
Stratification of contrast agent and polyethylene glycol in the thoracic aorta (see arrows) in a MIP (Maximum Intensity Projection –axial view; WL: 249, WW: 1455) reconstruction of a venous phase after injection of 1200 ml arterial and 1800 ml venous into the corpse (Group No 3)

The first mixing tests of PEG200 and the contrast agent Accupaque®300 showed that these two liquids only combined when they were very well mixed. If the PMCTA is not performed immediately after mixing, layering artefacts will occur in the vessels (Fig. 2 and Fig. 8). It is therefore important to ensure that the mixture is used immediately. It is also important to note that the contrast agent mixture loses intensity over time (Fig. 3). A possible explanation for this is that the contrast agent separates from the PEG and settles in the dorsal body sections due to gravity or leakage from the vessels into the environment. Figure 3 clearly shows that the measured Hounsfield units change over time and that the density in the body is lower than in the previous test in the tubes.

One of the main problems encountered in PMCTAs with PEG as a carrier substance was the same as described by Ross et al.: the visualization of the right coronary artery, which was not always contrasted due to the so-called layering artefact (Fig. 2). PMCTAs with PEG are therefore not recommended in cases where the evaluation of the right coronary artery is crucial.

In our study population, the mean BMI was 24.3 kg/m^2^ with a range of 19.7 to 39.1 kg/m^2^, although it should be noted that the second highest BMI was 30.4 kg/m^2^. Thus, there were very few bodies in the study sample that were underweight or significantly overweight. It is also possible that in small and/or underweight cadavers it may be needed to reduce the volume to avoid overfilling of the vasculature with possible leakage into surrounding tissues.

Further research is needed to test and adapt our proposed protocol for PMCTA performed on very small cadavers, e.g. children, and to determine whether there are other advantages of PEG as a carrier substance over paraffin oil in specific cases (e.g. child abuse, fat embolism).

In the future, it should also be investigated whether an adaptation of the reconstruction techniques, as already available for PMCTAs with oily contrast agent [27], can also be applied to PMCTAs with PEG-Accupaque.

However, even with the proposed protocol, overfilling in individual organs could not be completely prevented. It is not clear why the contrast agent leaked from the vascular system into the surrounding tissues in some cadavers and not in others. However, a possible explanation could be the post-mortem delay and internal decomposition, especially of the vessel walls. PEG and other water-soluble excipients have a lower viscosity than lipophilic excipients. This could explain the higher permeability of hydrosoluble vehicles such as PEG compared to lipophilic vehicles such as paraffin oil. This needs further investigation.

Further research should also include the influence of hydrosoluble fluids on the cadaver itself (tissues, vessels, organs) and whether they have a significant effect on post-mortem examinations such as toxicological screening and histopathological examinations. In addition, the influence of the contrast agent and the carrier substance PEG on the autopsy results, including possible artefacts, should be investigated.

## Conclusion and key results

The protocol presented demonstrates the simple and safe use of polyethylene glycol 200 as a carrier substance for the clinical iodinated contrast agent Accupaque® 300 and is applicable to adult cadavers. Such a protocol is necessary for the standardization of procedures and the comparison of different techniques, as well as for the defense of medico-legal cases in court. By using this protocol, very good image quality can be achieved and the relevant vascular structures can be assessed in a forensic setting. Only the right coronary artery was not opacified in about half of the cases due to a layer artefact in the ascending thoracic aorta. If such a question arises, for example in cases of suspected sudden cardiac death, a targeted PMCTA of the heart or a PMCTA with a different contrast agent mixture should be performed as an alternative [28, 29]. Furthermore, the possibility to have another standardized protocol in addition to the one already published by Grabherr et al. is an advantage, as the latter cannot be used in all cases (e.g. suspected fat embolism). However, further studies are needed to evaluate the influence of the contrast agent mixture on tissues and organs.

### Supplementary Information

Below is the link to the electronic supplementary material.

Supplementary file1 (PDF 371 KB)
