# Supplementary material for: Development of a protocol for standardized use of a water-soluble contrast agent with polyethylene glycol in post-mortem CT angiography
Source: Int J Legal Med. 2024 Apr 3;138(4):1437–46. doi: 10.1007/s00414-024-03218-y (PMC11164774; doi:10.1007/s00414-024-03218-y)
Supplement: Supplementary file 1 — Supplementary file1 (PDF 371 KB) [file 414_2024_3218_MOESM1_ESM.pdf]

Appendix 1

Group	No.	Phase	Sinus sag.	Sinus trans.	Vv. Jug.	Aa. Caro.	R ventricle	L ventricle	RCA	LCX	LAD	LPA	RPA	V. fem. R	A. fem. R	V. fem. L	A. fem. L	Aa. Brach.	Brain	Liver	Stomach	Spleen	Kidneys
1	1.1	art	3	2	3	1	3	3	1	1	1	3	3	3	1	3	1	1	1	3	2	3	1
		ven	1	2	1	1	2	3	1	1	1	1	1	1	1	1	1	1	1	1	1	3	1
		no dyn																					
1	1.2	art	3	2	1	1	2	2	1	1	1	1	1	1	1	1	1	1	1	1	3	3	1
		ven	2	2	1	1	1	2	1	1	1	1	1	1	1	1	1	1	1	1	1	1	1
		no dyn																					
2	2.1	art	1	1	1	1	3	3	3	1	1	3	3	2	1	2	1	1	1	2	2	3	1
		ven	1	1	1	1	2	3	3	1	1	1	1	1	1	1	1	1	1+	1+	2	1+	1+
		no dyn																					
3	3.1	art	3	1	2	1	3	2	3	1	1	3	3	2	1	3	1	1	1	1	1	1	1
		ven	1	1	1	1	2	1	3	1	1	1	1	1	1	1	1	1	1	1+	1+	2	1
		dyn	1	1	1	1	2	1	3	1	1	1	1	1	1	1	1	1	1	1+	1+	2	1
3	3.2	art	1	1	2	1	3	1	3	1	1	3	3	3	1	3	1	1	1	2	3	1	1
		ven	1	1	1	1	2	1	3	2	2	1	1	1	1	1	1	1	1	1+	1	1+	2
		dyn	1	1	1	1	2	1	1	1	1	1	1	1	1	1	1	1	1	1+	1	1+	2
3	3.3	art	1	2	2	1	3	2	3	1	1	3	3	3	1	3	1	1	2	2	1	1+	1+
		ven	1	1	1	1	3	3	3	2	2	1	1	1	1	1	1	1	2	1	1+	1+	1+
		dyn	1	1	1	1	3	2	3	1	1	1	1	1	1	1	1	1	2	1	1+	1+	1+
3	3.4	art	3	3	3	1	3	2	3	1	1	3	3	3	1	3	1	1	2	2	1	1	1
		ven	1	1	1	1	2	2	3	1	1	1	1	1	2	1	2	1	2	1+	1+	1	1
		dyn	2	1	1	1	2	3	3	1	1	1	1	2	1	1	1	1	2	1+	1+	1	1
3	3.5	art	1	1	1	1	2	2	3	1	1	3	3	3	1	3	1	1	1+	2	2	1+	1+
		ven	1	1	1	1	2	2	3	1	1	1	1	1	1	1	1	1	1+	1+	2	1+	1+
		dyn	1	1	1	1	2	2	3	1	1	1	1	2	1	1	1	1	1+	1+	2	1+	1+
3	3.6	art	3	3	1	1	3	1	1	1	1	3	3	3	1	3	1	1	1	1	1+	1	1
		ven	2	2	1	1	1	1	1	1	1	1	1	1	1	1	1	1	1	1	1+	1	1
		dyn	2	2	1	1	1	1	2	1	1	1	1	1	1	1	1	1	1	1	1+	1	1
3	3.7	art	3	3	3	3	3	3	3	3	3	3	3	3	1	3	3	3	3	1	3	3	1
		ven	3	3	3	3	3	3	3	3	3	3	3	1	1	3	3	3	3	1	3	3	1
		no dyn																					
4	4.1	art	3	3	3	1	3	1	3	1	1	3	3	3	1	3	1	1	1	1	1	1	1
		ven	3	3	1	1	2	1	3	1	1	1	1	1	1	1	1	1	1	1	1	1	1
		dyn	3	3	2	1	2	1	3	1	1	1	1	1	1	1	1	1	1	1	1	1	1
4	4.2	art	3	2	3	1	3	3	1	1	1	3	3	3	1	3	1	1	1	2	2	1	1+
		ven	3	2	1	1	2	2	2	1	1	1	1	1	1	1	1	1	1	1+	1+	1	1+
		dyn	3	2	2	1	3	2	3	1	1	1	1	1	1	1	1	1	1	1+	1+	1	1+
4	4.3	art	2	2	3	1	3	3	3	1	1	3	3	3	1	3	1	1	1	2	3	1	1
		ven	1	1	1	1	2	3	3	1	1	1	1	1	1	1	1	1	1	1	3	1	1
		dyn	1	1	1	1	2	3	3	1	1	1	1	1	1	1	1	1	1	1	3	1	1
4	4.4	art	3	3	3	1	3	2	3	1	1	3	3	3	1	3	1	1	2	2	3	1	1
		ven	1	2	1	1	2	1	2	2	1	1	1	1	1	1	1	1	2	1	2	1	1
		dyn	1	2	1	1	2	1	2	2	1	1	1	1	1	1	1	1	2	1	2	1	1

Appendix 1

Group	No.	Phase	Sinus sag.	Sinus trans.	Vv. Jug.	Aa. Caro.	R ventricle	L ventricle	RCA	LCX	LAD	LPA	RPA	V. fem. R	A. fem. R	V. fem. L	A. fem.	Aa. Brachi.	Brain	Liver	Stomach	Spleen	Kidneys
4	4.5	art	3	3	3	1	3	2	3	1	1	3	3	3	1	3	1	1	1	1	1	1	1+
		ven	1	1	1	1	2	1	3	1	1	1	1	1	1	1	1	1	1	1	1	1	1+
		dyn	1	1	1	1	2	1	3	1	1	1	1	1	1	1	1	1	1	1	1	1	1+
4	4.6	art	3	3	3	1	3	1	1	1	1	3	3	3	1	3	1	1	1	2	3	1	1
		ven	3	3	2	1	1	1	1	1	1	1	1	1	1	1	1	1	2	1	2	1	1
		dyn	3	3	2	1	1	1	1	1	1	1	1	1	1	1	1	1	2	1	2	1	1
4	4.7	art	3	3	3	2	3	2	3	2	2	3	3	3	1	3	1	2	3	3	3	3	3
		ven	3	3	1	1	2	2	3	2	2	1	1	1	1	1	1	1	3	1	3	3	1
		dyn	2	2	1	1	2	2	3	2	2	1	1	1	1	1	1	1	3	1	3	3	1
4	4.8	art	3	3	3	1	3	1	3	1	1	3	3	3	1	3	1	1	2	2	1	1	1
		ven	3	3	1	1	1	1	3	1	1	1	1	1	1	1	1	1	3	1	2	1	1
		dyn	3	3	1	1	1	1	3	1	1	1	1	2	1	1	1	1	3	1	2	1	1
4	4.9	art	3	3	3	1	2	1	1	1	1	3	3	3	2	3	1	1	2	1	2	3	1+
		ven	1	3	1	1	1	1	1	1	1	1	1	1	1	1	1	1	1	1+	2	3	1+
		dyn	1	2	1	1	1	1	1	1	1	1	1	1	1	1	1	1	1	1+	2	3	1+
4	4.10	art	3	3	3	2	3	1	3	1	1	3	3	3	1	3	1	3	1	1	3	2	1
		ven	3	3	3	3	1	1	3	1	1	1	1	1	1	1	1	3	3	1+	3	3	1
		dyn	3	3	3	2	1	1	3	1	1	1	1	1	1	1	1	3	3	1+	1	3	1
4	4.11	art	3	3	3	1	3	2	1	1	1	3	3	3	1	3	1	1	1	1	3	1	1
		ven	3	3	1	1	2	2	2	1	1	1	1	1	1	1	1	1	1	3	3	1+	1
		dyn	2	1	1	1	2	2	1	1	1	1	1	1	1	1	1	1	1	1	3	1+	1
4	4.12	art	3	3	2	1	3	1	1	1	1	3	3	3	1	3	1	1	1	3	3	1	1
		ven	1	1	1	1	1	1	1	1	1	1	1	1	1	1	1	1	1	1	3	1	1
		dyn	1	1	1	1	1	1	1	1	1	1	1	1	1	1	1	1	1	1	3	1	1
4	4.13	art	3	3	3	1	3	2	3	1	1	3	3	3	1	3	1	1	1	3	3	3	1
		ven	1	1	2	2	2	2	3	1	1	1	1	1	1	1	1	1	1	1	3	3	1
		dyn	1	1	1	1	2	1	3	1	1	1	1	1	1	1	1	1	1	1	3	3	1
